# Automatic detection of graticule isocenter and scale from kV and MV images

**DOI:** 10.1002/acm2.12558

**Published:** 2019-03-06

**Authors:** Raymond Fang, Jinzhong Yang, Weiliang Du, Laurence Court

**Affiliations:** ^1^ Department of Radiation Physics The University of Texas MD Anderson Cancer Center Houston TX USA; ^2^ Deparmtent of Physics and Astronomy Rice University Houston TX USA

**Keywords:** EPID, graticule, image processing, kV/MV films

## Abstract

**Purpose:**

To automate the detection of isocenter and scale of the mechanical graticule on kilo‐voltage (kV) or mega‐voltage (MV) films or electronic portal imaging device (EPID) images.

**Methods:**

We developed a robust image processing approach to automatically detect isocenter and scale of mechanical graticule from digitized kV or MV films and EPID images. After a series of preprocessing steps applied to the digital images, a combination of Hough transform and Radon transform was performed to detect the graticule axes and isocenter. The magnification of the graticule was automatically detected by solving an optimization problem using golden section search and parabolic interpolation algorithm. Tick marks of the graticule were then determined by extending from isocenter along the graticule axes with multiples of the magnification value. This approach was validated using 23 kV films, 26 MV films, and 91 EPID images in different anatomical sites (head‐and‐neck, thorax, and pelvis). Accuracy was measured by comparing computer detected results with manually selected results.

**Results:**

The proposed approach was robust for kV and MV films of varying image quality. The isocenter was detected within 1 mm for 98% of the images. The exceptions were three kV films where the graticule was not actually visible. Of all images with correct isocenter detection, 99% had a magnification detection error less than 1% and tick mark detection error less than 1 mm, with the exception of 1 kV film (magnification error: 3.17%; tick mark error: 1.29 mm) and 1 MV film (magnification error: 0.45%; tick mark error: 1.11 mm).

**Conclusion:**

We developed an approach to robustly and automatically detect graticule isocenter and scale from two‐dimensionla (2D) kV and MV films. This is a first step toward automated treatment planning based on 2D x‐ray images.

## 1 INTRODUCTION

In low‐ and middle‐income countries (LMICs), radiation therapy has been shown to be a cost‐effective therapy for many cancer treatments.^1^, ^2^ However, according to the IAEA‐DIRAC data,^3^ availability of radiation therapy in LMICs is extremely limited, and the shortage of radiation therapy staffing is significant.^4^, ^5^, ^6^ A recent report^7^ showed that by 2020, LMICs will have a deficit of around 12 000 radiation oncologists, 10 000 medical physicists, and 29 000 radiation therapy technologists. In response to the staffing shortage, automation of radiation treatment planning could potentially alleviate the staffing burden without compromising the quality of treatment in LMICs.^8^, ^9^, ^10^, ^11^

However, fully automated treatment planning is nontrivial. In particular, considering the resource limited settings in LMICs, many advanced technologies and equipment commonly available in the high resource setting countries are not available in LMICs. For example, three‐dimensional (3D) simulation based on CT images are common in many countries, but many clinics in LMICs do not have access to a CT scanner, or have to limit the number of patients for whom they perform CT imaging.^12^ Instead, conventional two‐dimensional (2D) simulation is used.^13^, ^14^ 2D treatment simulation images are typically taken using radiographic film, which are then viewed on a light box. In some settings, this has been replaced with flat panel x‐ray detectors, but many centers still use film. Furthermore, in situations where the x‐ray tube is out of service, it may be necessary to use mega‐voltage (MV) imaging [using the radiation beams from the radiotherapy treatment device, and film or Electronic Portal Imaging Device (EPID)].

To use the radiographic films for automatic treatment planning, the first step is to digitize them. Traditionally this is done using a specialized film digitizer. Other options are available in resource scarce environments, such as using an inexpensive commercial flatbed document scanner,^15^ although care should be taken such the digitized image does not distort the film.

Important steps in treatment planning using portal images typically include determination of patient treatment position, beam geometry, treatment isocenter, field limits, contours, and dosage.^10^, ^16^ For isocentric treatment, determining the isocenter of treatment field is a key step. The intended treatment isocenter is the primary reference location for radiation treatments and typically needs to be determined at the beginning of treatment planning to facilitate the subsequent planning tasks such as defining beam geometry and dose calculation.^17^

In 2D simulation, the intended treatment isocenter can be determined by using a mechanical graticule when taking the 2D simulation images. The mechanical graticule is visible in the images and its isocenter can surrogate the intended treatment isocenter. To automate the treatment planning, it is necessary to automatically identify the isocenter and scale of the graticule shown on the 2D simulation images, which are likely to be created using a kilo‐voltage (kV) x‐ray tube and film, but could be created using the mega‐voltage (MV) x rays from a linac or cobalt unit also, as described above. Inspired by the linac quality assurance (QA) of localizing the isocenter of radiation fields described by Du et al.^18^, we proposed an automated process by combining template matching,^19^ Hough transform,^20^ and Radon transform^21^ to detect the isocenter and scales of graticule from kV/MV films. We tested our proposed automated detection algorithm on both scanned kV and MV films and digital MV images obtained from EPID. In this study, we showed the feasibility to automate the process of localizing isocenters and determining scales from kV/MV films with mechanical graticule, which will allow the automation of 2D radiation treatment planning.

## 2 MATERIALS AND METHODS

### 2.A Patient data

**Figure 1:**
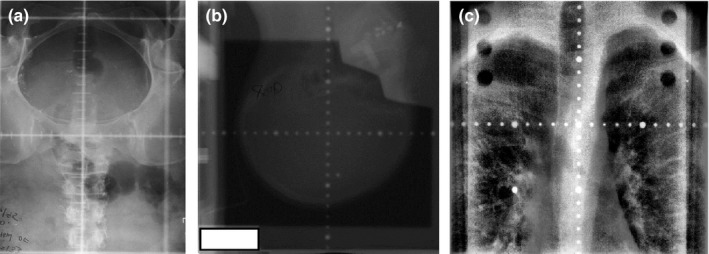
Example of (a) a KV film, (b) a MV film, and (c) an EPID image used in this study.

Under the approval of our institutional review board, 23 kV films, 26 MV films, and 91 digital EPID images of cancer patients in different anatomical sites including head and neck, thorax, abdomen, and pelvis were obtained for this study. The kV films have the graticules with cross‐shaped tick marks, while the MV films and the EPID images have the graticules with dotted tick marks. Both kV films and MV films were scanned with a specialized film digitizer and converted into gray scale images with a dots‐per‐inch (DPI) of 50. Examples of the scanned kV and MV films and the EPID images were shown in Fig. 1. Most scanned kV films have poor image quality, representing the most challenging cases in automatic detection of the graticules.

### 2.B Image preprocessing

**Figure 2:**
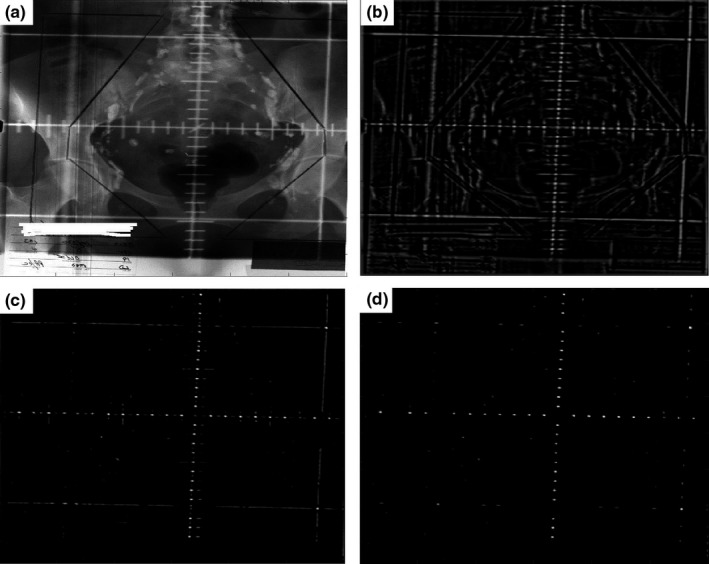
Preprocessing of graticule images. Entire preprocessing includes 3 steps applied sequentially to the original kV film: template correction; linear weighting; and non‐maxima suppression. (a) The original kV film image (I); (b) the processed image after template correlation (C); (c) the processed image after linear weighting (C′); (d) the processed image after non‐maxima suppression (C″).

Appropriate preprocessing on the original images should be done before the automatic isocenter detection and graticule magnification determination. The preprocessing was separated into 3 steps: perform correlation with a template; apply a linear weighting to correlated image; and perform nonmaxima suppression. The entire preprocessing steps are shown in Fig. 2, and the details of each step are described as below.

#### 2.B.1 Template correlation

**Figure 3:**
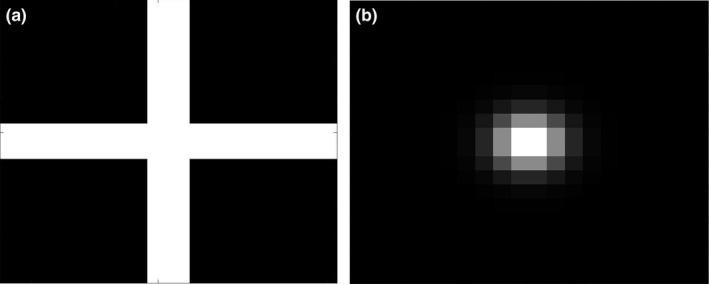
Generic templates for (a) kV films and (b) MV films and electronic portal imaging device images with a magnification factor of 1.

The first step was to perform a correlation with a predefined template for the scanned images.^19^ The correlation step found pixels on the scanned image that might be part of tick marks. Let *I* denote the scanned image and *T* the template, which was designed to detect the tick marks in the graticule. Based on the shape of the tick marks (crosses for kV films and dots for MV images), binary templates were created (Fig. 3). In the case that a graticule has tick marks of other shape, a different template matching the tick mark can be created as well and the algorithm can be tweaked for the new template. Both templates had a size of 1 cm × 1 cm with a pixel resolution of 50 DPI. In the cross‐shaped template, the foreground value was 1 and the background value was 0, and the width of the two axis of the cross‐shaped template was set to 1.2 mm based on the actual thickness of graticule axes. Letting (*x*_0_, *y*_0_) denote the center of the template and *w* the width of template, the intensity of the template was modeled with

(1) $$T\left(x,y\right)=\left\{\begin{array}{cc} 1, & x-x_{0}>\frac{w}{2}\;or\;y-y_{0}>\frac{w}{2}\\ 0, & \mathrm{else} \end{array}\right..$$

For the circular template, the intensity of the template had a radial Gaussian weight with a standard deviation (SD) of $\frac{1}{12}$ mm, which was determined using the radius of the circles of graticule tick marks so that two SDs of the Gaussian equals to the radius of the circles. It was noted that the best results occurred when the width and radii of the templates corresponded to the width and radii of the graticule tick marks. Letting *r* denote the SD, $\frac{1}{12}$ in this case, the intensity of the template was modeled with

(2) $$T\left(x,y\right)=\exp\left[-\frac{{\left(\left(x,y\right)-\left(x_{0},y_{0}\right)\right)}^{2}}{2r^{2}}\right].$$

The normalized cross‐correlation (NCC)^22^ values were computed for each pixel in the scanned image by correlating with the template as:

(3) $$NCC\left(u,v,T\right)=\frac{\sum_{x,y}\left[I\left(x,y\right)-\bar{I_{u,v}}\right]\left[T(x-u,y-v)-\overset{¯}{T}\right]}{{\sqrt{\sum_{x,y}\left[I\left(x,y\right)-\bar{I_{u,v}}\right]}}^{2}\sum_{x,y}{\left[T(x-u,y-v)-\overset{¯}{T}\right]}^{2}}$$

where $\overset{¯}{T}$ was the mean intensity value of the template T and $\bar{I_{u,v}}$ was the mean intensity value of the image portion overlaid with the template. In order to account for magnification, the templates were scaled by 1.1, 1.3, and 1.5, respectively, in the consideration of the actual magnification of the graticule in the range of [1.0, 1.5].^23^ The maximum correlation value from these three templates was chosen. Mathematically, let *m* be the magnification factor and *T*_*m*_ be the template of a magnification *m*. The output of template correlation, *C*(*x*,* y*), was:

(4) $$C\left(x,y\right)=\max_{m=\{1.1,1.3,1.5\}}NCC\left(x,y,T_{m}\right)$$

where $T_{m}\left(x-x_{0},y-y_{0}\right)=T\left(\frac{x-x_{0}}{m},\frac{y-y_{0}}{m}\right)$, with (*x*_0_, *y*_0_) the center of the template.

#### 2.B.2 Linear weighting

The following linear weighting was applied to *C*(*x*,* y*):

(5) $$C^{\prime }\left(x,y\right)=\max\left(0,\frac{C\left(x,y\right)-0.5C_{\max}}{0.5C_{\max}}\right)$$

here, *C*_*max*_ was the largest correlation value in C. The linear weighting process filtered out most of the pixels that did not belong to tick marks. The assumption of the linear weighting was that the tick marks had higher correlation values with the template than the rest of the scanned image. In addition, this process also set a threshold for a pixel to be considered as a tick mark as the half of the overall maximum correlation value *C*_*max*_, and linearly rescaled correlation values to be above this threshold.

#### 2.B.3 Non‐maxima suppression

Non‐maxima suppression was performed to remove lines on the film that were not graticule axis.^24^ The assumption was that the graticule axes should have the largest value after the correlation process. Let C″ denote the image after non‐maxima suppression process, *D* the physical distance in centimeter between two nearest tick marks, and dpcm (dots per centimeter) the resolution of the scanned image (dpcm = DPI/2.54). The first step of the non‐maxima suppression was to remove points which did not likely represent tick marks:

(6) $$C^{\prime \prime }\left(x,y\right)=\left\{\begin{array}{cc} C^{\prime }\left(x,y\right) & if\;C\left(x,y\right)>\left(C(x+i,y+j)\right)\forall -\frac{D}{\mathrm{dpcm}}\leq i,j\leq \frac{D}{\mathrm{dpcm}}\\ 0 & \mathrm{else} \end{array}\right..$$

The second step was to restore pixel values at the locations neighboring the nonzero values in C″ to those in C′. The neighborhood was defined to be within a Euclidean distance of 3 pixels:

(7) $$C^{\prime \prime }\left(x,y\right)=\left\{\begin{array}{cc} C^{\prime }\left(x,y\right)\;if\;\exists i,j\;such\;that\;C^{\prime }\left(x+i,y+j\right)>0, & i^{2}+j^{2}+3^{2}\\ 0, & \mathrm{else} \end{array}\right..$$

This step ensured that enough nonzero pixels remained in C″ for the subsequent Hough transform and Radon transform to detect graticule axial lines.

### 2.C Isocenter detection

Detecting the treatment isocenter is equivalent to detecting the graticule axes. The intended treatment isocenter is the intersection of the two graticule axes. In general, the two graticule axes are perpendicular to each other and are aligned analogously to the *x* and *y* axis on a typical graph. We refer to the axis aligned roughly parallel to the *x*‐axis on a graph as the horizontal axis and axis aligned roughly parallel to the *y*‐axis on a graph as the vertical axis.

A combination of the Hough transform^20^ and Radon transform^21^ was applied to the preprocessed image C″ to detect the vertical and horizontal axes of the graticule. The Hough transform was used to determine possible parameterizations of the lines representing the graticule axes. For detection of lines, the Hough transform used the following parametric representation, with *θ* representing the angle counterclockwise from the *x*‐axis:

(8) ρ=xcosθ+ysinθ.

Every nonzero pixel (*x*,* y*) in C″ was transformed to the Hough space (*ρ*,* θ*) using the Hough transform. To detect the horizontal axis, *θ* were restricted to 85^∘^ ≤ *θ* ≤ 95^∘^. Let *N*(*ρ*,* θ*) denote the number of nonzero pixels (*x*,* y*) in C″ that were parameterized with (*ρ*,* θ*). Let (*ρ*_*m*_, *θ*_*m*_) denote the parameters having largest *N*(*ρ*,* θ*) and (*ρ*_*m*2_, *θ*_*m*2_) the parameters corresponding to the second largest value of *N*(*ρ*,* θ*). If $N\left(\rho_{m},\theta_{m}\right)>2N\left(\rho_{m2},\theta_{m2}\right)$, the parameters (*ρ*_*m*_, *θ*_*m*_) were chosen to parameterize the horizontal graticule axis. Otherwise, the parameters (*ρ*,* θ*) that maximized the line integral *R*(*ρ*,* θ*) of the line represented by xcosθ+ysinθ in C″ was chosen to parameterize the horizontal axis:

(9) $$R\left(\rho ,\theta \right)=\int_{-\infty }^{\infty }C^{\prime \prime }\left(\rho cos\theta -zsin\theta ,\rho sin\theta +zcos\theta \right)dz.$$

To improve the speed, we used only the parameter sets of (*ρ*,* θ*) corresponding to the largest four values of *N*(*ρ*,* θ*) for the above line integral. For 85° ≤ *θ* ≤ 95° degrees, values of *ρ*_*h*_ and *θ*_*h*_ that maximize *R*(*ρ*,* θ*) were used to parameterize the horizontal axis of the graticule. This procedure was equivalent to the Radon transform^21^ except that only a small set of possible (*ρ*, *θ*) values were considered in order to reduce the computational time.

**Figure 4:**
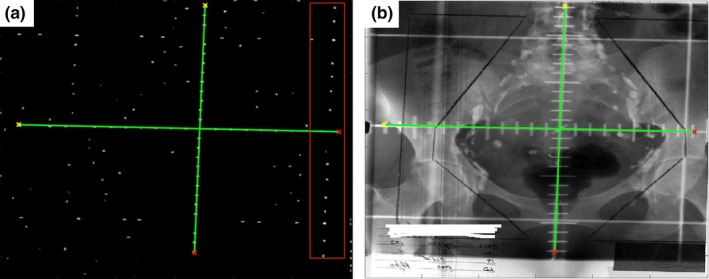
Graticule axes and isocenter detection. The graticule axes were found by using a combination of Hough transform and Radon transform. (a) Automatically detected graticule axes overlaid on the preprocessed image. Points in red box could be detected as vertical axis using Hough transform, but the Radon transform correctly filtered it out. (b) Automatically detected graticule axes and isocenter overlaid on the original image.

The vertical axis of the graticule was detected using the similar procedure for horizontal axis except that the *θ* value was restricted to [−5°, 5°]. The intersection of the vertical and horizontal axes then defined the treatment isocenter, noted as (*x*_*iso*_, *y*_*iso*_). The graticule axes and isocenter detection is exemplified in Fig. 4.

### 2.D Magnification and tick mark detection

Once the isocenter was identified, we were able to detect the tick marks from the preprocessed image C″. It is known that the physical distance between two nearest graticule tick marks is 1 cm, and this distance showing on the film is normally greater than 1 cm because of magnification during imaging. The magnification is determined by the distance between the physical graticule and the imaging plane, varying for different films and normally with a value between 1.1 and 1.5.^23^ Unlike the EPIDs, the magnification factor is normally unavailable for a film. However, appropriate processing on the scanned films is able to recover the magnification, as described below.

The tick mark points of the graticule are determined by extending from isocenter along the graticule axes multiples of the magnification value. Let *V*_*m*_ and *H*_*m*_ be the set of tick mark points at a magnification of *m* forming the vertical axis and horizontal axis, respectively, and *P*_*m*_ = *V*_*m*_ ∪ *H*_*m*_, the collection of tick mark points. The coordinates of the tick mark points can be represented as

(10) $$\begin{array}{cc} V_{m} & =\left\{\left(x,y\right)\in R^{2}|\rho_{v}=x\;cos\theta_{v}+y\;sin\theta_{v};y\right.\\ & \left.=y_{\mathrm{iso}}+j*m*\text{dpcm}*\cos\left(\theta_{v}\right),\forall j\in Z;y_{\min}\leq y\leq y_{\max}\right\}; \end{array}$$

(11) $$\begin{array}{cc} H_{m} & =\left\{\left(x,y\right)\in R^{2}|\rho_{h}=x\;cos\theta_{h}+y\;sin\theta_{h};\;x\right.\\ & \left.=x_{\mathrm{iso}}+j*m*\text{dpcm}*\sin\left(\theta_{h}\right),\forall j\in Z;x_{\min}\leq x\leq x_{\max}\right\}. \end{array}$$

where (*ρ*_*v*_, *θ*_*v*_) and (*ρ*_*h*_, *θ*_*h*_) are parameters spanning the graticule vertical and horizontal lines, and $\left[y_{\min},y_{\max}\right]$ and [*x*_min_, *x*_max_] are the extreme point coordinates for graticule vertical and horizontal axes, respectively. The problem of finding the coordinates of the tick marks therefore reduces to determining the correct magnification value.

The tick mark points determined by Eqs. (10) and (11) were used to select pixels from the processed image C″. A correct magnification value *m* should give a maximum average intensity value of all selected pixels. Formally, the optimal magnification, *M*_*c*_, was determined using the following optimization equation:

(12) $$M_{c}=\max_{m\in R}f\left(m\right)=\max_{m\in R}\frac{1}{|P_{m}|}\sum_{\forall \left(x,y\right)\in P_{m}}C^{\prime \prime }\left(P_{m}\right)$$

where |*P*_*m*_| is the number of points in *P*_*m*_. Initial guess of the optimal magnification was done by sampling the value of *m* between 1 and 1.5 with an incremental of 0.01, which maximized Eq. (12). This step ensured that the following maximization algorithm would not converge to a poor local maximum. The initial guess was then used to initialize the golden section search and parabolic interpolation algorithm^25^ (fminbnb function in Matlab, Mathworks, Natick, MA) to determine the optimum magnification *M*_*c*_.

Once the optimal magnification was determined, we performed the following step to further optimize the isocenter location. The assumption here was that the optimal isocenter location should give the best detection of tick marks. We assumed that small perturbations (*x* ^′^, *y* ^′^) added to the isocenter $\left(x_{\mathrm{iso}},y_{\mathrm{iso}}\right)$ could bring it to an optimal location, (*X*_*iso*_, *Y*_*iso*_), that is, $\left(X_{\mathrm{iso}},Y_{\mathrm{iso}}\right)=\left(x_{\mathrm{iso}},y_{\mathrm{iso}}\right)+\left(x^{\prime },y^{\prime }\right)$. Following a similar procedure in determining the optimal magnification, an optimal solution of (*x* ^′^, *y* ^′^) was determined using the following optimization function:

(13) $$\begin{array}{cc} V_{M}^{\prime } & =\left\{\left(x,y\right)\in R^{2}|\rho_{v}=x\;cos\theta_{v}+y\;sin\theta_{v};y\right.\\ & \left.=y^{\prime }+y_{\mathrm{iso}}+j*M*\text{dpcm}*\cos\left(\theta_{v}\right),\forall j\in Z;y_{\min}\leq y\leq y_{\max}\right\}; \end{array}$$

(14) $$\begin{array}{cc} H_{M}^{\prime } & =\left\{\left(x,y\right)\in R^{2}|\rho_{h}=x\;cos\theta_{h}+y\;sin\theta_{h};x\right.\\ & \left.=x^{\prime }+x_{\mathrm{iso}}+j*M*\text{dpcm}*\sin\left(\theta_{h}\right),\;\forall j\in Z;x_{\min}\leq x\leq x_{\max}\right\}; \end{array}$$

(15) $$\left(x_{s},y_{s}\right)=\max_{\left(x^{\prime },y^{\prime }\right)}\sum_{\forall \left(x,y\right)\in V_{M}^{\prime }\cup H_{M}^{\prime }}C^{\prime \prime }\left(V_{M}^{\prime }\cup H_{M}^{\prime }\right).$$

**Figure 5:**
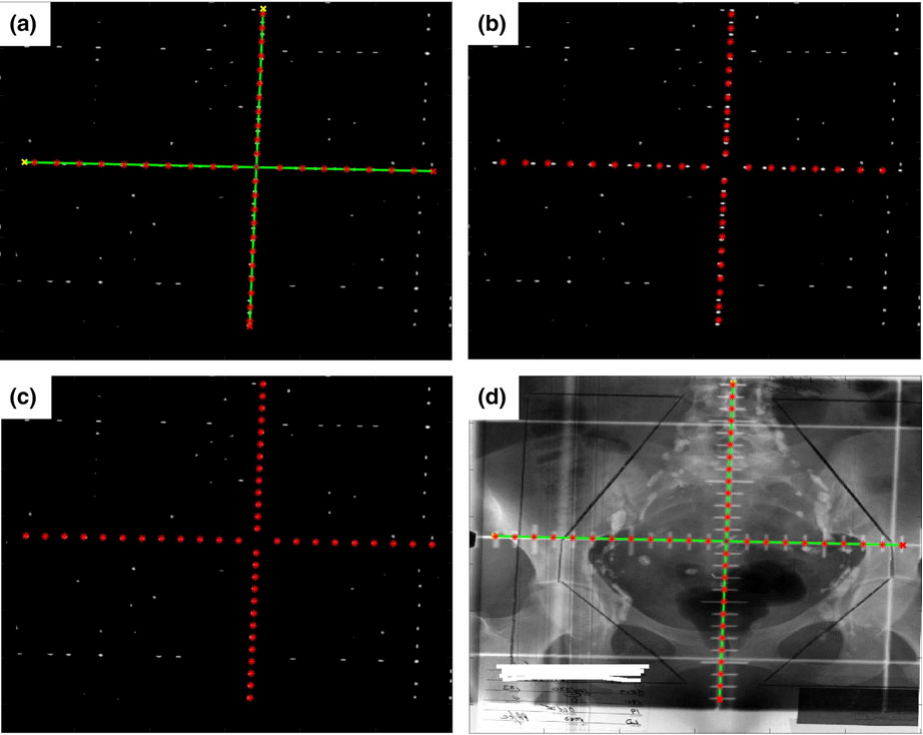
Magnification and tick mark detection. (a) The coordinates of tick marks (including isocenter) are restricted to lie on the graticule axes being equidistant from each other. (b) Calculated tick mark points for a given non‐optimal magnification value overlaid on the preprocessed image C″. (c) Calculated tick mark points with optimal magnification value overlaid on the preprocessed image C″. (d) Detected tick mark points, isocenter, and graticule axes overlaid on the original image.

The initial guess of (*x* ^′^, *y* ^′^) was set to (0, 0) when the fminbnb function in Matlab was used to find the optimal solution of Eq. (15). The final optimal isocenter location was $\left(X_{\mathrm{iso}},Y_{\mathrm{iso}}\right)=\left(x_{\mathrm{iso}},y_{\mathrm{iso}}\right)+\left(x_{s},y_{s}\right)$. Once the optimal isocenter location and magnification value were determined, the tick marks were automatically determined by extending from isocenter along the graticule axes multiples of the magnification value, as shown in Fig. 5.

### 2.E Approach validation

For validation, the automatically detected isocenter and tick mark locations were compared with corresponding manually selected points. The manual selection was done on the original scans. When a point was selected, the image was zoomed in the local region for accurate selection. The selection was performed for isocenter and each tick mark on horizontal and vertical axes. The isocenter was selected three times in this process.

The actual magnification of the image, *M*_*m*_, was estimated from the manually selected points as follows. The distance between two nearest tick marks was 1 cm in reality; therefore, the distance between two nearest tick marks on the image was equivalent to the magnification of the image and this distance was estimated by the following equation:

(16) $$M_{m}=\frac{\max_{h_{1},h_{2}\in H_{m}}\left(d(h_{1},h_{2})\right)\max_{v_{1},v_{2}\in v_{m}}\left(d(v_{1},v_{2})\right)}{n-2},$$

where *d*(·,·) represented the Euclidean distance of two points measured from the image, *H*_*m*_ and *V*_*m*_ the collection of manually selected tick mark points on the horizontal and vertical axes, respectively, and *n* the total number of points in *H*_*m*_ and *V*_*m*_. Note that the isocenter was in both *H*_*m*_ and *V*_*m*_ so it was counted twice here. The error of automatically detected magnification was represented in percentage as $\frac{M_{c}-M_{m}}{M_{m}}*100\%$, where *M*_*c*_ was the automatic detection magnification described in Section 2.D.

The isocenter was manually selected three times. The mean distance of any two selected points was used to quantify the intra‐observer variability in selecting the isocenter, and used to compare with the isocenter detection error, which was defined as the Euclidean distance between the automatically detected isocenter and the geometric mean of the manually selected isocenters. The tick mark detection error was found by calculating the Euclidean distance between the automatically detected tick mark and the corresponding manually selected tick mark. The median of all tick mark errors in one graticule axis was used as the representative value of that axis. For each image, two tick mark errors were reported, one for the horizontal axis and one for the vertical axis. Both the isocenter error and tick mark error were scaled by dividing the magnification factor *M*_*m*_ to reflect the actual physical error. In our automatic detection algorithm, we assumed that the tick marks were evenly spaced along graticule axes. However, of the images under testing, 11 MV films had uneven physical spacing between tick marks so that our automatic magnification and tick mark detection algorithm does not work. For these 11 films, only the isocenter detection error was evaluated.

## 3 RESULTS

**Figure 6:**
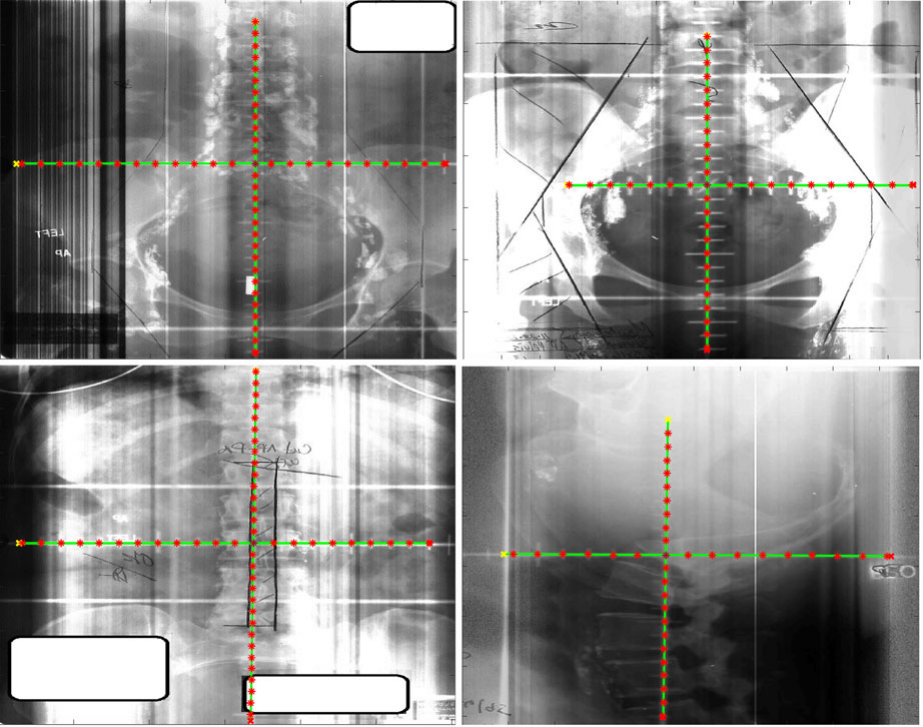
Illustration of automatic detection of graticule including isocenter and tick marks from kV film scans of varying image quality.

**Figure 7:**
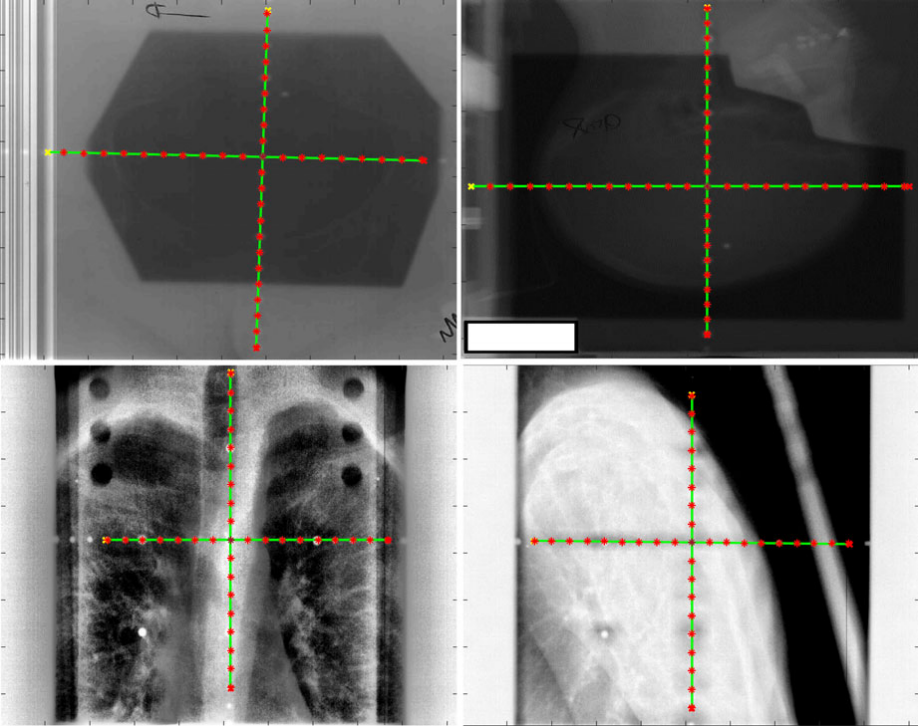
Illustration of automatic detection of graticule including isocenter and tick marks from MV scans (top row) and electronic portal imaging device images (bottom row).

The proposed approach was tested on the 23 scanned kV films, 26 scanned MV films, and 91 EPID images. Overall, the processing time was fast. The typical runtime, including preprocessing, isocenter detection, magnification estimation, and tick mark detection, was approximately 0.2 s for an image of 800 × 1000 pixels on a computer with 2 GHz Intel Core i7 CPU and 8 GB memory. Figures 6 and 7 show some examples of automatically detected graticules on kV films, MV films, and EPID images. Quantitative evaluation results are summarized in Table 1, with details described as below.

**Table 1:** Summary of quantitative results (mean ± SD) of the isocenter detection error, intra‐observer variability, magnification detection error, and tick mark detection error

	Isocenter error (mm)	Intra‐observer variability (mm)	Magnification error (%)	Median tick mark error (mm)
kV films (*n* = 20)	0.3 ± 0.2	0.3 ± 0.2	0.36 ± 0.69	0.4 ± 0.2
MV films (*n* = 26)	0.4 ± 0.2	0.3 ± 0.1	0.22 ± 0.15	0.6 ± 0.2
EPID images (*n* = 91)	0.2 ± 0.1	0.2 ± 0.1	0.18 ± 0.13	0.2 ± 0.1

### 3.A Isocenter detection

**Figure 8:**
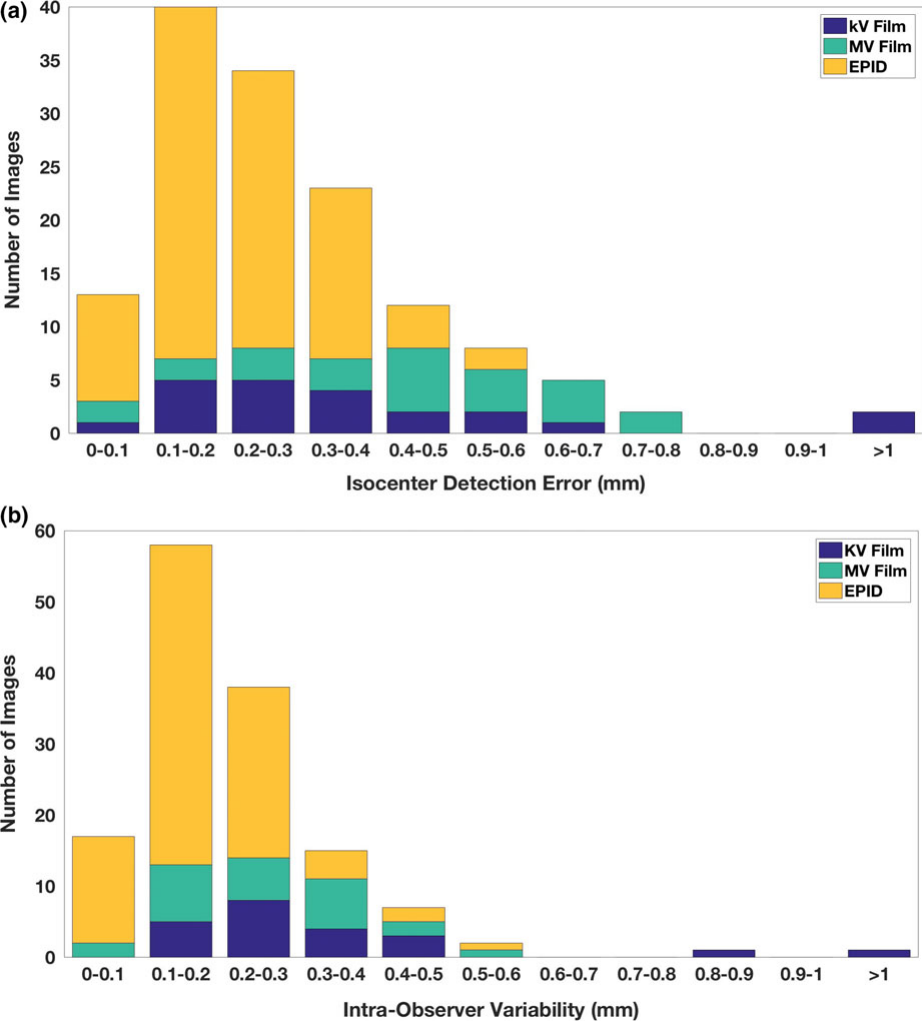
Histogram of (a) isocenter detection error and (b) intra‐observer variability in selecting the isocenters. All kV films, MV films, and electronic portal imaging device images except three kV films have isocenter detection error less than 1 mm. The isocenter of the three kV films with an error > 1 mm cannot be manually selected accurately as well.

**Figure 9:**
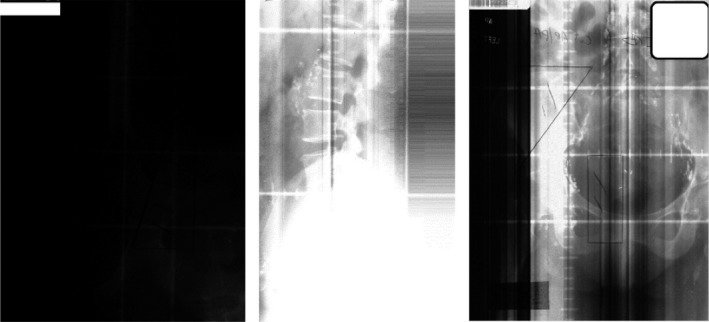
Illustration of three kV films where the isocenter could not be automatically detected. These three films have very poor image quality and the graticule is not actually visible on the scanned image.

The proposed approach was robust to kV and MV films of varying image quality. The isocenter could be detected in most images despite varied complexities such as occlusion and glare. The histogram of isocenter detection error is shown in Fig. 8(a), compared with the intra‐observer variability shown in Fig. 8(b). The isocenter was detected with accuracy less than 1 mm for all but three kV films where the graticule was not actually visible. These three kV films were illustrated in Fig. 9. Because the isocenter could not be automatically detected, these three cases were not included in the subsequent analysis.

The average isocenter detection errors for kV films (excluding the aforementioned three kV films), MV films, and EPIDs were 0.3 ± 0.2 mm, 0.4 ± 0.2 mm, and 0.2 ± 0.1 mm, respectively. For comparison, the corresponding intra‐observer variability in manually selecting the isocenter was 0.3 ± 0.2 mm, 0.3 ± 0.1 mm, and 0.2 ± 0.1 mm, respectively, as shown in Table 1. The computer detection error was comparable to the intra‐observer variability, which showed that the automatic isocenter detection had a similar performance of manual isocenter placement. Of all cases with successful isocenter detection, the maximum error was 0.8 mm for KV films, 0.8 mm for MV films, and 0.5 mm for EPID images.

### 3.B Magnification and tick mark detection

**Figure 10:**
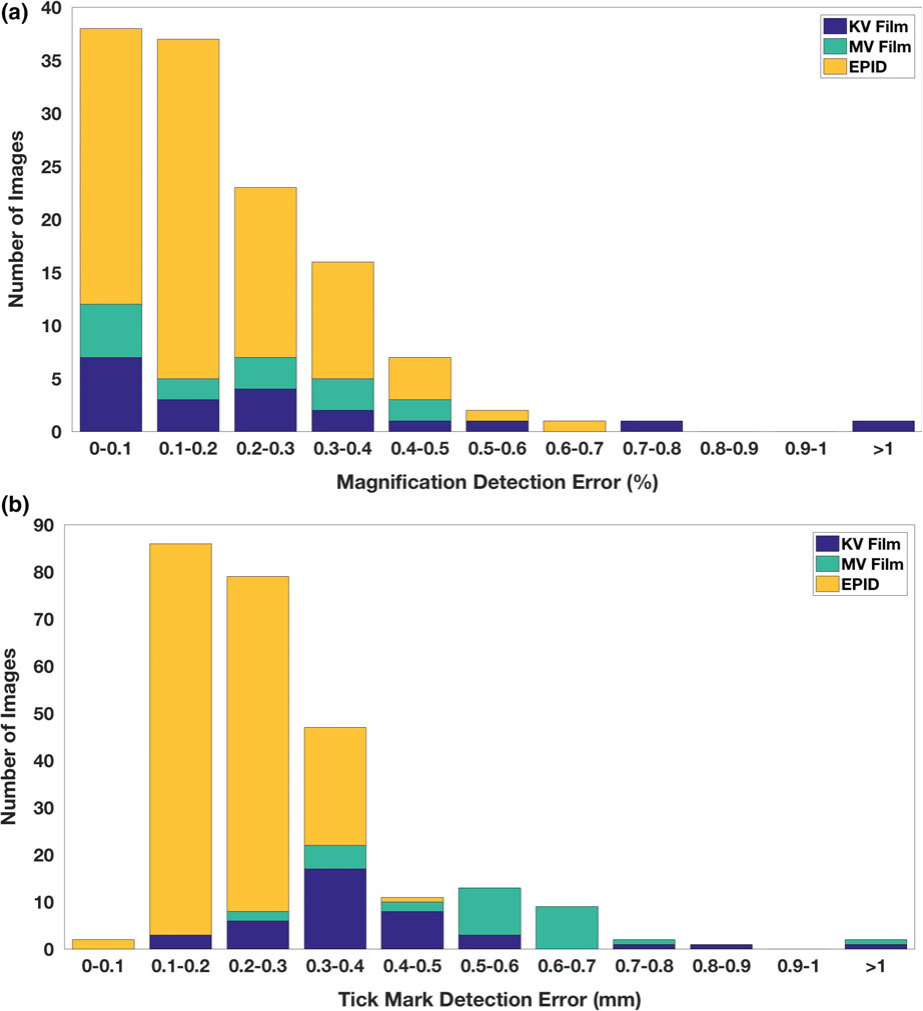
Histogram of (a) magnification detection error and (b) tick mark detection error. The median of all tick mark errors in one graticule axis was presented and for each image two tick mark errors were reported.

Magnification and tick mark detection were applied to all those images with successful isocenter detection. The histograms of magnification and tick mark detection error are shown in Fig. 10. Of all images under evaluation, 99% had a magnification detection error less than 1% with the exception of one kV film, which had an error of 3.17%. The mean magnification error for kV films, MV films, and EPID images were 0.29%, 0.22%, and 0.18%, respectively (Table 1). This result showed that the magnification could be automatically detected from images very accurately, no matter what images were scanned from kV or MV films, or were obtained through EPID.

**Figure 11:**
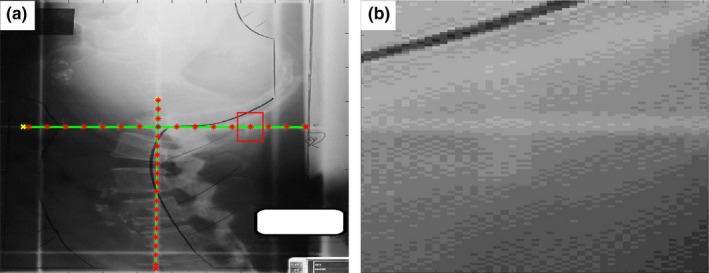
(a) The kV film with the worst magnification detection error (3.17%). (b) The zoomed portion of the image (a) in red box. The lack of contrast between the tick marks and the background led to a suboptimal tick‐mark detection.

Tick mark detection error was small as well for all images under evaluation. Ninety‐nine percent of the images had a tick mark detection error <1 mm with the exception of one kV film and one MV film. The kV film, which had a magnification detection error of 3.17%, had a tick mark error of 1.3 mm for the horizontal axis. The error was due to the poor image quality. Many of the graticule tick marks in the image had very low contrast to the background, resulting to ineffective preprocessing for the automatic detection (Fig. 11). On the other hand, the MV film, which had a magnification detection error of 0.45%, had a tick mark error of 1.1 mm for the vertical axis. The mean tick mark detection error for kV films, MV films, and EPID images were 0.4, 0.6, and 0.2 mm, respectively (Table 1). These results showed that the tick mark detection was very accurate. Upon examination of some kV scans, we noticed that a slight asymmetry of the distance of nearest tick marks to the left and right of the isocenter were often observed. This was the most significant factor contributing to the tick mark detection error. The isocenter refinement technique described in Section 2.A could reduce the impact from the asymmetry.

## 4 DISCUSSION

The proposed approach for determining graticule isocenter and tick marks was very robust to a variety of conditions. This was evidenced in the low isocenter detection errors and tick mark detection errors in kV films, MV films and EPID images of different image quality and under varied imaging conditions. In addition, we compared the automatic detection results with intra‐observer variability in manually selecting the isocenters. The automated process was shown to be comparable with human beings in manual selection the isocenters. In all images under evaluation, the kV films were the most complicated due to poor image quality. Factors including glare, occlusion, and low contrast made the automatic graticule detection extremely difficult. Unknown magnification and rotational positioning during film scan further complicated the automatic detection. Yet, the proposed approach succeeded in detecting the graticule for nearly all cases.

Notice that all EPID images had very good detection accuracy and results were better than either kV or MV films. EPID images were acquired digitally without the need of scan, which had much less uncertainty and the image quality was much better than the kV and MV films. Our proposed approach worked very well with all 91 EPID images. While the digital x‐ray image acquisition becomes more accessible, the use of films becomes less. This implies that our proposed approach will be more robust when the image quality is no longer a concern.

As aforementioned, the conventional 2D simulation based on x‐ray images is still the choice of many LMICs due to the limited resources.^12^, ^13^, ^14^ The shortage of qualified staff for radiation treatment planning also presents challenge in delivering high quality radiation treatment in LMICs.^7^ Our study has shown that isocenter on 2D x‐ray images can be automatically detected by computers and automated process was comparable to manual selection conducted by human beings. This automated process will facilitate the development of automated treatment planning based on 2D x‐ray images, which potentially can address the issue of staff shortage in LMICs. This signifies an important application of our study in LMICs. On the other hand, the proposed approach can also facilitate the linac QA^26^, ^27^ by reducing the workload in analyzing the QA images for LMICs.

Our study has some limitations. One major limitation is the assumption that the tick marks were evenly spaced along graticule axes. For some x‐ray films, there was geometric distortion during imaging so that this assumption was not true. In our study, the tick marks on eleven MV films could not be correctly detected due to this reason. Nevertheless, this issue did not present on the digitally acquired EPID images, which will be more accessible in the future. In addition, though the automatic graticule detection works very well, it has not been integrated into an automated treatment planning workflow for verification. As such, this work is a pilot study to verify the feasibility. Its clinical usability needs further validation. This will be our future study.

## 5 CONCLUSION

We have developed an image processing approach to automatically and robustly detect graticule isocenter and tick marks from 2D x‐ray images. Our results showed that the automated process was comparable to manual selection conducted by human beings. This essentially allows the automation of treatment planning based on 2D x‐ray images. Together with automated treatment planning, this technique will have important applications in LMICs.

## CONFLICT OF INTEREST

The authors declare no conflict of interest.

## References

[1] Barton MB , Frommer M , Shafiq J . Role of radiotherapy in cancer control in low‐income and middle‐income countries. Lancet Oncol. 2006;7:584–595. 16814210 10.1016/S1470-2045(06)70759-8

[2] Van Der Giessen P‐H , Alert J , Badri C , et al. Multinational assessment of some operational costs of teletherapy. Radiother Oncol. 2004;71:347–355. 15172152 10.1016/j.radonc.2004.02.021

[3] IAEA‐DIRAC . IAEA‐DIRAC: Availability of Radiation Therapy; 2018; https://dirac.iaea.org/Query/Map2?mapId=0. Accessed April 13, 2018.

[4] Fisher BJ , Daugherty LC , Einck JP , et al. Radiation oncology in Africa: improving access to cancer care on the African continent. Int J Radiat Oncol Biol Phys. 2014;89:458–461. 24929154 10.1016/j.ijrobp.2013.12.032

[5] Jaffray DA , Gospodarowicz M . Bringing global access to radiation therapy: time for a change in approach. Int J Radiat Oncol Biol Phys. 2014;89:446–447. 24929153 10.1016/j.ijrobp.2014.05.019

[6] McCarroll R , Youssef B , Beadle B , et al. Model for estimating power and downtime effects on teletherapy units in low‐resource settings. J Global Oncol. 2017;3:563–571. 10.1200/JGO.2016.005306 PMC5646876 29094096

[7] Datta NR , Samiei M , Bodis S . Radiation therapy infrastructure and human resources in low‐ and middle‐income countries: present status and projections for 2020. Int J Radiat Oncol Biol Phys. 2014;89:448–457. 24751411 10.1016/j.ijrobp.2014.03.002

[8] Beadle BM , McCarroll R , Kisling K , et al. Development of 3D automated radiation treatment planning for low‐ and middle‐income countries. Int J Radiat Oncol Biol Phys. 2017;99:E637–E637.

[9] Olsen LA , Robinson CG , He GR , et al. Automated radiation therapy treatment plan workflow using a commercial application programming interface. Pract Radiat Oncol. 2014;4:358–367. 25407855 10.1016/j.prro.2013.11.007

[10] Court LE , Kisling K , McCarroll R , et al. Radiation planning assistant ‐ a streamlined, fully automated radiotherapy treatment planning system. J Vis Exp. 2018;134:e57411. 10.3791/57411 PMC5933447 29708544

[11] Yang J , Chu D , Dong L , Court LE . Advantages of simulating thoracic cancer patients in an upright position. Pract Radiat Oncol. 2014;4:e53–e58. 24621432 10.1016/j.prro.2013.04.005

[12] Van Dyk J , Zubizarreta E , Lievens Y . Cost evaluation to optimise radiation therapy implementation in different income settings: a time‐driven activity‐based analysis. Radiother Oncol. 2017;125:178–185. 28947098 10.1016/j.radonc.2017.08.021

[13] Mutrikah N , Winarno H , Amalia T , Djakaria M . Conventional and conformal technique of external beam radiotherapy in locally advanced cervical cancer: dose distribution, tumor response, and side effects. J Phys. 2017;884:012122.

[14] Rodin D , Grover S , Xu MJ , et al. Radiotherapeutic management of non‐small cell lung cancer in the minimal resource setting. J Thorac Oncol. 2016;11:21–29. 26762736 10.1016/j.jtho.2015.09.008

[15] Matney JE , Parker BC , Neck DW , Henkelmann G , Rosen II . Evaluation of a commercial flatbed document scanner and radiographic film scanner for radiochromic EBT film dosimetry. J Appl Clin Med Phys. 2010;11:3165. 20592699 10.1120/jacmp.v11i2.3165 PMC5719943

[16] Podgoršak EB , International Atomic Energy Agency . Radiation Oncology Physics: A Handbook for Teachers and Students. Vienna: International Atomic Energy Agency; 2005.

[17] Halabi T , Faddegon B . Practical quantitative measurement of graticule misalignment relative to collimator axis of rotation. J Appl Clin Med Phys. 2010;11:3318. 21081892 10.1120/jacmp.v11i4.3318 PMC5720415

[18] Du WL , Yang J , Luo DS , Martel M . A simple method to quantify the coincidence between portal image graticules and radiation field centers or radiation isocenter. Med Phys. 2010;37:2256–2263. 20527559 10.1118/1.3397452

[19] Brunelli R . Template Matching Techniques in Computer Vision: Theory and Practice. Chichester: Wiley; 2009.

[20] Du W , Yang J . A robust Hough transform algorithm for determining the radiation centers of circular and rectangular fields with subpixel accuracy. Phys Med Biol. 2009;54:555. 19124954 10.1088/0031-9155/54/3/006

[21] Deans SR . The Radon Transform and Some of Its Applications. Mineola: Dover Publications; 2007.

[22] Lewis JP . Fast normalized cross‐correlation. Vis Inter. 1995;10:120–123.

[23] Herman MG , Balter JM , Jaffray DA , et al. Clinical use of electronic portal imaging: report of AAPM Radiation Therapy Committee Task Group 58. Med Phys. 2001;28:712–737. 11393467 10.1118/1.1368128

[24] Neubeck A , Gool LV . Efficient non‐maximum suppression. In: 18th International Conference on Pattern Recognition (ICPR'06); 2006.

[25] Forsythe GE , Malcolm MA , Moler CB . Computer Methods for Mathematical Computations. Englewood Cliffs, NJ: Prentice Hall; 1976.

[26] Klein EE , Hanley J , Bayouth J , et al. Task Group 142 report: quality assurance of medical accelerators. Med Phys. 2009;36:4197–4212. 19810494 10.1118/1.3190392

[27] Rowshanfarzad P , Sabet M , O'Connor DJ , Greer PB . Isocenter verification for linac‐based stereotactic radiation therapy: review of principles and techniques. J Appl Clin Med Phys. 2011;12:185–195. 10.1120/jacmp.v12i4.3645 PMC5718736 22089022

